# Acceptor–Donor Molecular Heterojunction Control of π‑Orbital-Induced Magnetic Properties of a 3d Ferromagnet

**DOI:** 10.1021/acs.nanolett.5c03762

**Published:** 2025-08-20

**Authors:** Servet Ozdemir, Matthew Rogers, Zabeada Aslam, Mannan Ali, Gilberto Teobaldi, Timothy Moorsom, B. J. Hickey, Oscar Cespedes

**Affiliations:** † School of Physics and Astronomy, 4468 University of Leeds, Leeds LS2 9JT, United Kingdom; ‡ School of Chemical and Process Engineering, 4468 University of Leeds, Leeds LS2 9JT, United Kingdom; § Scientific Computing Department, Science and Technology Facilities Council (STFC), UK Research and Innovation (UKRI), Rutherford Appleton Laboratory, Didcot OX11 0QX, United Kingdom

**Keywords:** spinterface, electric field effect, acceptor−donor heterojunction, exchange bias, magnetic hardening

## Abstract

Metal–organic molecule interfaces have given rise to a wide range of magnetic phenomena. These effects arise due to spin-polarized charge transfer and enhanced exchange interaction at metallo-molecular hybridization sites, where tunability via electric fields beyond ferroelectric interfaces remains to be demonstrated. Here, we explore manipulating the magnetism of cobalt with the intrinsic electric field generated at C_60_/phthalocyanine heterojunctions, a combination commonly used in organic photovoltaics. The results give evidence for a C_60_ layer thickness-dependent control of hybridization effects on cobalt. We find that the heterojunctions may attenuate the hybridization effects, with changes in coercivity and magnetization due to the built-in electric field. An emergent exchange bias is attributed to an enhanced Rashba interaction for thicker C_60_ layers. Our study clarifies some of the questions in the field of molecular “spinterface” physics and demonstrates that internal electric field generation is a promising method for manipulation of metallo-molecular interfaces up to room temperature.

## Graphical abstract

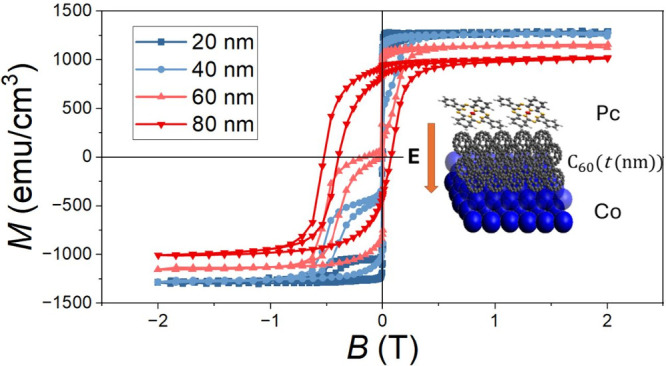

Fundamental research on magnetism has underpinned much of the development behind information technology, and it will remain to be at least equally important with the emergence of neuromorphic and functional artificial intelligence systems.^1^ Reducing the size of information storage and memory systems to interfaces with minimized energy consumption are necessary steps for both the miniaturization and reduced power usage goals of the industry, with the latter being expected to grow to 20% of the total global energy output by 2030.^2^ Organic molecules have been shown to be promising sustainable systems at interfaces, exhibiting a wide range of spin-dependent interfacial phenomena when hybridized to various materials.^3–5^ A major goal of the field of molecular spintronics has been the electric field control of interfacial effects,^6–8^ which, as well as being key in novel device concepts, is necessary for a better fundamental understanding, for example, of spin physics at metallo-molecular interfaces.^9–11^ Puzzling results have been obtained in these systems, such as the observation of antiferromagnetic ordering persisting to room temperature in ultrathin MnPc films interfaced with Co, leading to the interpretation of asymmetric out-of-center hysteresis loops for Co as an exchange bias effect.^12^ Since then, further work has been carried out to clarify the observed effects,^10^ including studies of the Co/MnPc interface with a Cu space layer^13^ as well as metalloporphyrin^14^ and non-magnetic C_60_ interfaces with Co.^9,11^

Here, we first carry out a comparative study between magnetic pinning and hardening effects at Co interfaces with non-magnetic H_2_Pc and C_60_, and also with CuPc, a molecule that is magnetic at low temperatures when grown templated.^15^ It has been suggested that electric fields could be used to tune these molecular interface effects,^7^ but difficulties to grow oxide layers on molecular films without degradation or contamination and the metalization of the molecular interface make it difficult to achieve a voltage gating with conventional dielectrics. In this work, we report the growth of molecular acceptor–donor junctions on top of the metallo-molecular interface to obtain an electric field effect. We use fullerene (C_60_) and phthalocyanine molecules (CuPc, MnPc, and H_2_Pc), a well-known material combination for efficient photovoltaic systems.^16^ The molecular heterojunction generates an intrinsic electric field extending to the metallo-molecular interface, which enables the manipulation of interface effects. Furthermore, a systematic study of the effect of the molecular heterojunction on the metallo-molecular interface contributes to unraveling the role of spin-polarized charge transfer in the magnetic properties of metallo-molecular interfaces.

We compare H_2_Pc–, C_60_–, and CuPc–Co interfaces grown on Pt seed layers (see the Supporting Information for more detail on growth and characterization, including 4D STEM). The structure of each molecule is depicted in Figure 1a (i–iii). A comparative study allows us to determine relevant parameters for the physical mechanism behind modified magnetic properties of the metallo-molecular interface. All molecules contain π orbitals and carbon hexagon–pentagon units as common features. Unlike CuPc, which may show intrinsic magnetic order at low temperatures,^15^ H_2_Pc and C_60_ are diamagnetic and do not contain any metal ion. As shown in previous studies, a strong magnetic hardening effect is expected in Co thin films with molecular interfaces due to enhanced exchange coupling via π–3d orbital hybridization.^9,17^ Figure 1a (iv) shows a schematic of the spin-polarized density of states for both Co and the Co/molecule interface post-chemisorption.^6,18^ The schematic depicts the presence of spin-polarized charge transfer to the interface, where the sign of the spins can be manipulated by cooling the sample in a magnetic field with the desired polarity.

**Figure 1:**
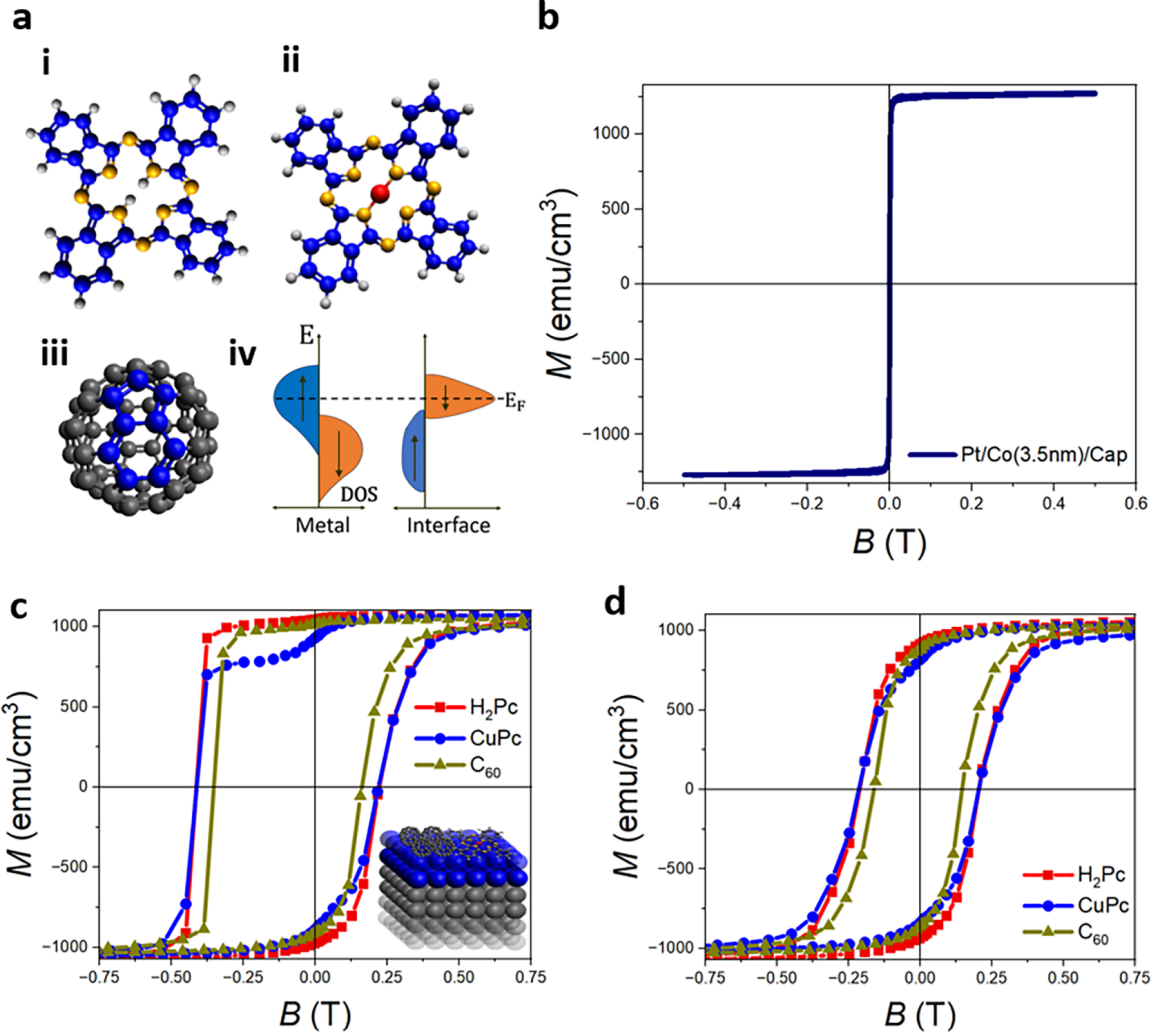
Interfacial magnetic pinning and magnetic hardening effects on metal and metal-free phthalocyanines hybridized with cobalt. (a) Schematics depicting the structure of (i) hydrogen phthalocyanine, (ii) copper phthalocyanine, (iii) C_60_ molecules, with hexagon–pentagon units common to all molecules depicted in blue, and (iv) spin-polarized density of states depicting the origin of magnetic pinning and the hardening at the cobalt/organic molecule interface. (b) Magnetic hysteresis curve measured on a reference Pt/Co (3.5 nm)/metal-capped interface measured at *T* = 10 K. (c) Magnetic hysteresis curve measured on capped Pt/Co (3.5 nm) interfaces (see the inset schematic) of 20 nm thick metal-free hydrogen phthalocyanine (red), copper phthalocyanine (blue), and C_60_ (olive) post-field cooling with 2 T at *T* = 10 K, suggesting magnetic pinning. (d) Training curves measured at hydrogen phthalocyanine (red), copper phthalocyanine (blue), and C_60_ (olive) interfaces depicting magnetic hardening and suggesting the absence of any exchange bias field measured at *T* = 10 K.

The capped reference control films Pt/Co (3.5 nm) remain magnetically soft down to 10 K, with a coercivity of <10 mT, as shown in Figure 1b. The saturation magnetization is measured to be ≈1275 emu/cm^3^, deviating from the bulk value but in agreement with thin-film measurements.^9,19^ The initial magnetization loops measured at Pt/Co (3.5 nm)/molecular multilayers are shown in Figure 1c, with the molecular film being C_60_ (20 nm), CuPc (20 nm), or H_2_Pc (20 nm), and measured at 10 K post-field cooling. These multilayers with molecular interfaces reveal reduction in magnetization and a coercivity of 0.35–0.4 T on the first magnetic reversal, large compared to control Co films due to spin-polarized charge transfer from the metal to the organic molecule,^9^ with data for H_2_Pc in red, CuPc in blue, and C_60_ in olive green. The coercivity drops to 0.15–0.2 T at the second and subsequent magnetization reversal points (Figure 1d) due to a training effect. We presume that this is due to an irreversible change of the spin order at the interface after the initial reversal. The change in coercivity between the first and subsequent reversals make the initial hysteresis loop appear to be asymmetric. The very similar magnetic hysteresis curves measured on all systems (identical for H_2_Pc and CuPc interfaces), including the training effect and disappearance of an asymmetry after the first reversal, suggest a magnetic pinning effect that is not linked to intrinsic magnetic properties of the molecules but is rather due to π-orbital hybridization with Co d orbitals.^11^ Exchange bias post-training loops has been measured for example on ferromagnet–spin glass coupled frustrated systems.^20^ It has been recently reported that magneto-molecular interfaces can also give rise to a novel glassy state, which may be responsible for the training effect.^21^

Spectroscopic studies on C_60_/CuPc interfaces provide evidence that an interface dipole^18–20^ is responsible for the n–p behavior of the junction.^22,23^ The mechanism behind the interface dipole formation has been however subject to debate in work with molecular or polymer n–p junction interfaces.^24^ Mechanisms based on both charge transfer^25^ and the formation of interface states^26,27^ have been proposed. More recently, a mechanism based on anisotropic charge distribution and associated Coulomb repulsion at the interface has been suggested.^28,29^ This last mechanism is applicable to C_60_/CuPc heterostructures,^22^ with the interface dipole being always negative going from C_60_ to CuPc, independently of their growth order.^23^ CuPc molecules in C_60_/CuPc interfaces were found to lead to charge repulsion at the interface, in both face-on and edge-on configurations, leading to an excess charge of up to 0.023 e^–^ per interface C_60_ molecule. These electrostatic dynamics have been interpreted as the origin of the interface dipole at C_60_/CuPc interfaces.^22^

We utilize this dipole-generated, built-in electric field at the C_60_/CuPc (CuPc/C_60_) bilayers to modify the hybridization, magnetic hardening, and pinning at Co/C_60_ (Co/CuPc) interfaces underneath. The magnetic properties observed for the Co (3.5 nm)/C_60_ (20 nm) interface (Figure 2a) is now modulated by the distance between the metallo-molecular interface and the molecular junction dipole (i.e., the distance between the Co surface and the C_60_/Pc interface). The schematic of the energy level alignment depicting the formation of the interfacial dipoles is depicted in Figure 2b, where we note the value of the interface dipole at the C_60_/CuPc interface, Δ_2_, measured to be in the range from −0.22 to −0.27 eV.^22^ The value of the dipole at the Co/C_60_ interface, Δ_1_, is computed to be 0.1 eV (see the Supporting Information for more detail on DFT calculations). The magnetometry curves for Co/C_60_ (*t* nm)/CuPc (20 nm) interfaces grown on a Pt (4 nm) seed layer are shown in Figure 2c, where the thickness of the C_60_ layers is 10, 20, and 50 nm for each of the respective curves. It is striking that both the magnetic hardening and pinning effects can be attenuated by the additional C_60_/CuPc interface.

**Figure 2:**
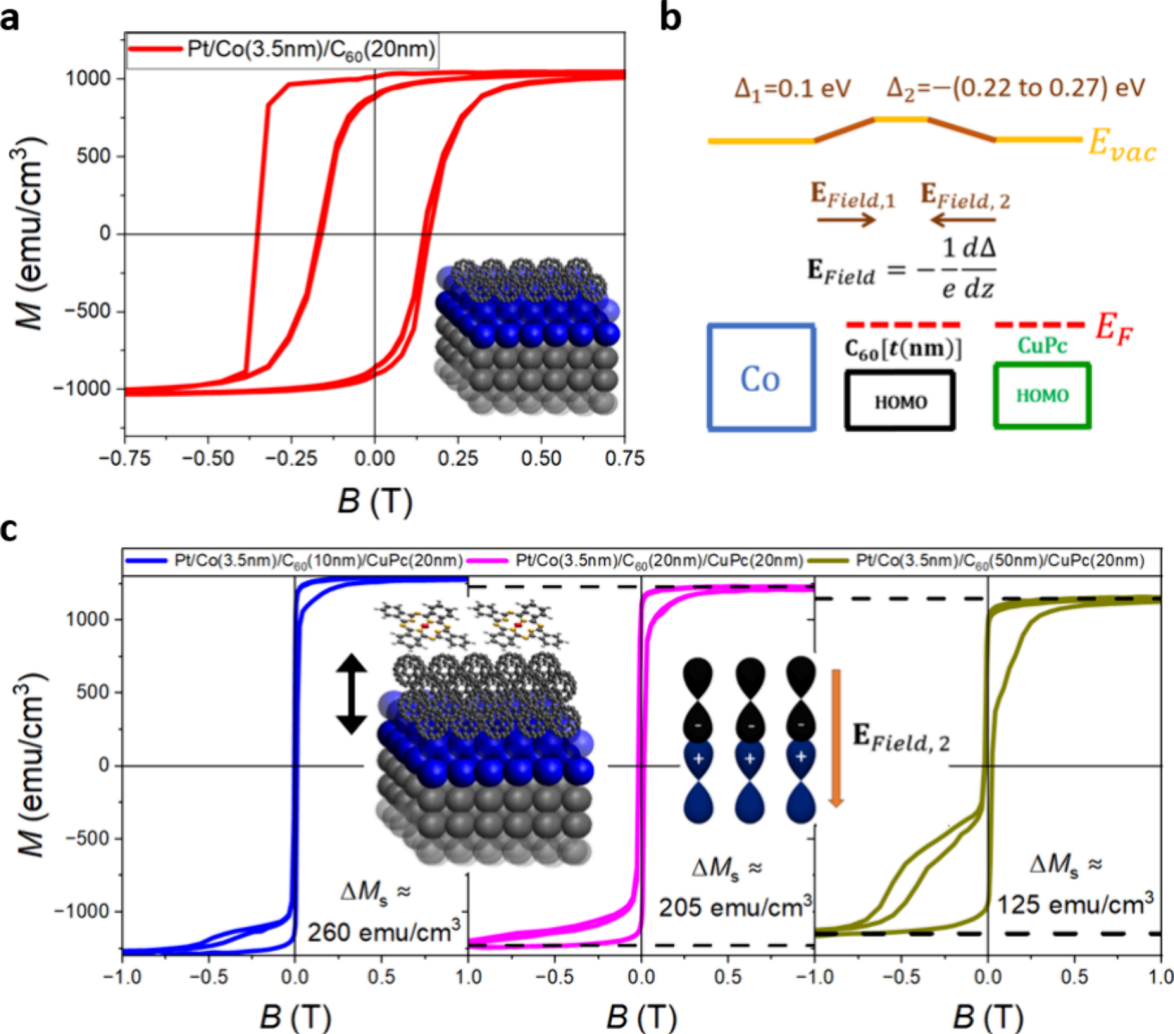
Built-in electric field effect on a Co/C_60_ metallo-molecular interface at *T* = 10 K. (a) Magnetic hysteresis curve measured on a capped Pt/Co (3.5 nm)/C_60_ (20 nm) structure, illustrating both magnetic pinning (initial curve measured after field cooling) and hardening effects (training curve). (b) Schematic illustrating energy dynamics at Co/C_60_/CuPc multilayers with electric fields due to dipoles of opposite signs emerging at each interface, Co/C_60_ and C_60_/CuPc. (c) Magnetic hysteresis curves measured on capped Pt/Co (3.5 nm)/C_60_ (*t* nm)/CuPc (20 nm) structures with the C_60_ layer thickness *t* = 10 nm (blue left), 20 nm (pink middle), and 50 nm (green right). The softer loop with only part of the sample switching at high fields strongly suggests an attenuation of the Co/C_60_ interfacial hybridization effects by the electric field generated at the C_60_/CuPc interface. The inset schematics show the sample structure (left) and the electric field induced at the molecular heterojunction acting on the metallo-molecular interface (right). Further results for other molecular heterojunctions are shown in Figures S6 and S7.

The electric dipole has an effect not only on the shape of the loop but also on the sample magnetization. The saturation magnetization is ≈1275 emu/cm^3^ for the sample without molecules Pt/Co (3.5 nm); see Figure 1b. This value then drops to ≈1000 emu/cm^3^ in Pt/Co (3.5 nm)/C_60_ (20 nm) without a CuPc heterojunction, which we take to be our reference value (Δ*M*_S_ = 0). However, the magnetization of Pt/Co (3.5 nm)/C_60_ (10 nm)/CuPc (20 nm) is almost the same as the control Pt/Co (3.5 nm) sample without molecules due to the generated electric field at the molecular heterojunction; *M*_S_ ≈ 1260 emu/cm^3^ (Δ*M*_S_ ≈ 260 emu/cm^3^); see Figures 2c and 3. The magnetization then drops progressively as the C_60_ thickness “*t*” is increased, as shown in Figure 3. The change in magnetization can be fit by an exponential decay curve. This dependence of the change in magnetization with the C_60_ layer thickness confirms the presence of an expected Debye screening of the electric field within fullerenes due to their intrinsic carrier density.^30,31^ The electric field at the CuPc interface is due to an excess interface charge of approximately 0.023 e^–^ per C_60_ molecule, leading finite sheet density at the C_60_/CuPc interface.^22^ The calculated screened electric field is shown in the inset of Figure 3 (see the Supporting Information for more details). The magnetization reaches the reference value of the sample without CuPc when *t* = 200 nm; i.e. *M*_S_ ≈ 1000 emu/cm^3^ for Pt/Co (3.5 nm)/C_60_ (200 nm)/CuPc (20 nm) (see Figure S5 of the Supporting Information). This provides evidence that the electric field attenuates the hybridization at the Co/C_60_ interface. When the C_60_ layer is 200 nm thick, the distance to the organic heterojunction is so large that the electric field at the metallo-molecular interface is negligible; therefore, the presence of the CuPc layer does not affect the Co–C_60_ coupling. We find that this attenuation effect is not just limited to Pt/Co/C_60_/CuPc structures but is also present on Pt/Co/C_60_/MnPc and Pt/Co/C_60_/H_2_Pc multilayers (see Figures S6 and S7). In the case of an opposite polarity interface dipole, i.e., a Pt/Co (3.5 nm)/CuPc (*t* nm)/C_60_ (20 nm) structure where there is a net electric field pointing in the opposite direction, we find that the first magnetic reversal coercivity is enhanced from a reference value from ≈0.4 to ≈0.5 T (see Figure S8a).

**Figure 3:**
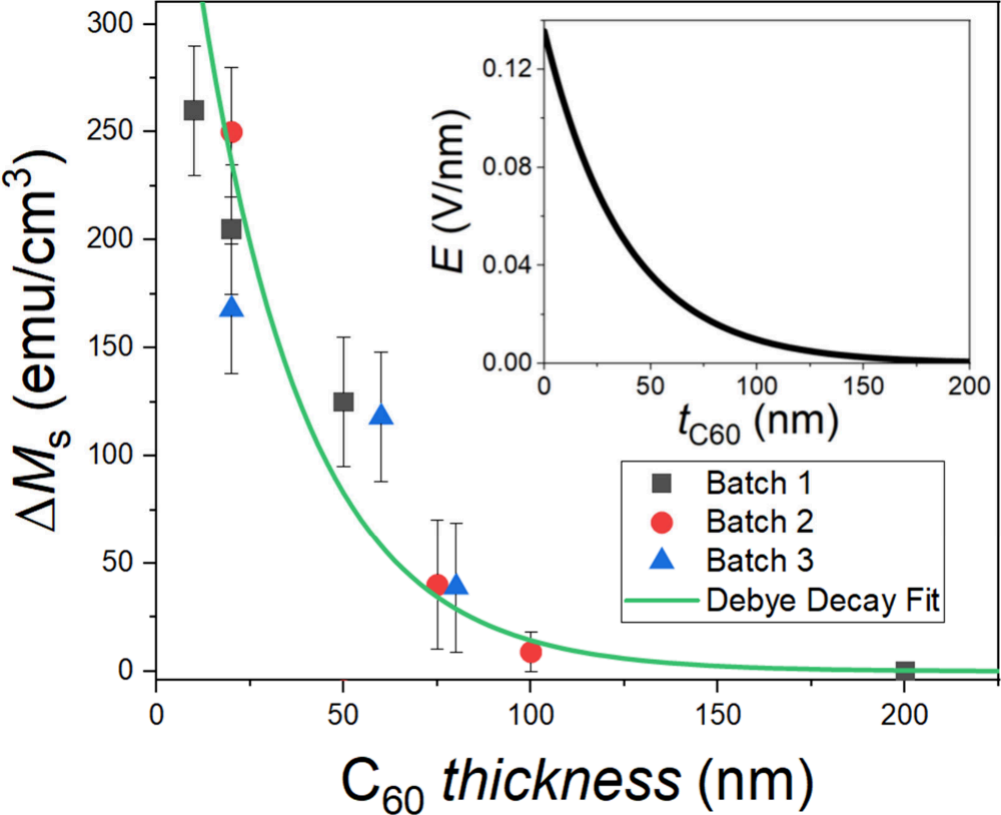
Debye-screening-induced exponentially decaying change in saturation magnetization on Co interfaces with C_60_ (*t* nm)/CuPc (20 nm) acceptor–donor heterojunctions. The figure shows the change in measured saturation magnetization measured at *T* = 10 K on capped Pt/Co (3.5 nm)/C_60_ (*t* nm)/CuPc (20 nm) structures as a function in the C_60_ layer thickness where samples from different batches are indicated using different symbols, with the green fit being an exponential decay fit implied by the presence of Debye screening. The calculated decaying electric field generated at the C_60_/CuPc interface as a function of the C_60_ thickness is shown in the inset.

During magnetometry measurements of Pt/Co (3.5 nm)/C_60_ (*t* nm)/CuPc (20 nm) as well as structures where the CuPc layer was replaced with either MnPc or H_2_Pc molecules, we have come across samples that exhibit exchange bias; see Figure 4 and Figures S6 and S7. The saturation magnetization values measured on these samples with exchange bias deviate from the Debye screening trend indicated in Figure 3, where the saturation magnetization is reduced compared to Pt/Co/C_60_ (20 nm) films without a fullerene–phthalocyanine heterojunction. We attribute the exchange bias emergence to an enhanced Rashba coupling due to a finite electric field (≈0.01–0.05 V/nm) on the Co/C_60_ interface of the Pt/Co (3.5 nm)/C_60_/CuPc heterostructure, where the π-orbital hybridization persists on thicker C_60_ film interfaces due to the electric field being significantly smaller. A recent study of exchange bias has predicted that, in the event of an enhanced Rashba coupling constant at a magnetic interface, an in-plane electric polarization emerges with an associated electric field. This field can generate exchange bias as well as a magnetization change.^32^ Metallo-molecular interfaces have already demonstrated enhanced spin–orbit coupling effects on Pt interfaces.^33^ The emerging exchange bias, accompanied by a reduced magnetization, could then be due to an increase in the Rashba coupling at the Co/C_60_ interface induced by the dipolar electric field. The exchange bias measured on first, second, and third batches was found to be ≈85, ≈53, and ≈43 mT, respectively. For an inverted molecular donor–acceptor structure of Pt/Co (3.5 nm)/CuPc (*t* nm)/C_60_ (20 nm), we also find an exchange bias offset to be present, in this case for all thicknesses of CuPc up to 100 nm, with the maximum bias being ≈67 mT measured for a 10 nm thick CuPc interface (see Figure S8b). The magnetic bias emerging in these inverted structures at *T* = 10 K is likely to be due to the electric field pointing in the opposite direction, which will enhance π–d orbital hybridization at the interface. It has been previously demonstrated that spins in Cu ions in flat-lying CuPc are pointing out of plane.^15,34^ Interestingly, an exchange bias emergence in such a case, where the interfacial spins and electric field are pointing along the same direction, has also been discussed theoretically^32^ to explain exchange bias results on STO/LAO/LSMO interfaces.^35^ We also note that we have seen further exchange bias emergence as well as reduction in magnetization on Nb-capped samples due to degradation (measured 6 months after growth; see Figure S9 of the Supporting Information). Similar degradation effects have also been recently reported on Au-capped samples.^36^

**Figure 4:**
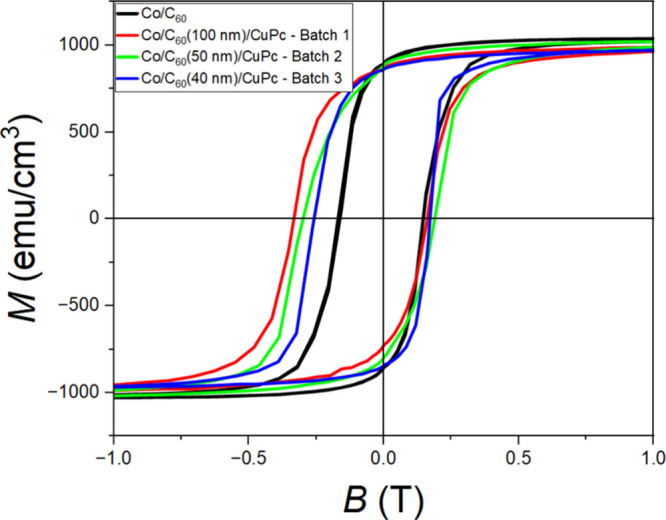
Post-field cooling training loops measured at *T* = 10 K on a capped Pt/Co (3.5 nm)/C_60_ (black curve) interface and Pt/Co (3.5 nm)/C_60_ (*t* nm)/CuPc (20 nm) samples of different batches, 1, 2, and 3, where the respective thicknesses of C_60_ layers are 100, 50, and 40 nm, with the shift of the center of the hysteresis loops at respective values of ≈85, ≈53, and ≈43 mT clearly suggesting exchange bias emergence on Pt/Co (3.5 nm)/C_60_ (*t* nm)/CuPc (20 nm) samples.

Although these low-temperature results are key to understanding metallo-molecular interfaces, practical applications benefit from easily measurable room-temperature effects. Magnetization loops measured at 300 K for varying C_60_ thickness in Pt/Co (3.5 nm)/C_60_ (*t* = 10–200 nm)/CuPc (20 nm) heterostructures as well as a reference Pt/Co (3.5 nm)/C_60_ (20 nm) interface are shown in Figure 5a and b. It is evident that the electric field generated at the molecular C_60_/CuPc (acceptor–donor) junction has a strong influence on the room-temperature coercivity of Co interfaces with C_60_ molecules (Figure 5c).

**Figure 5:**
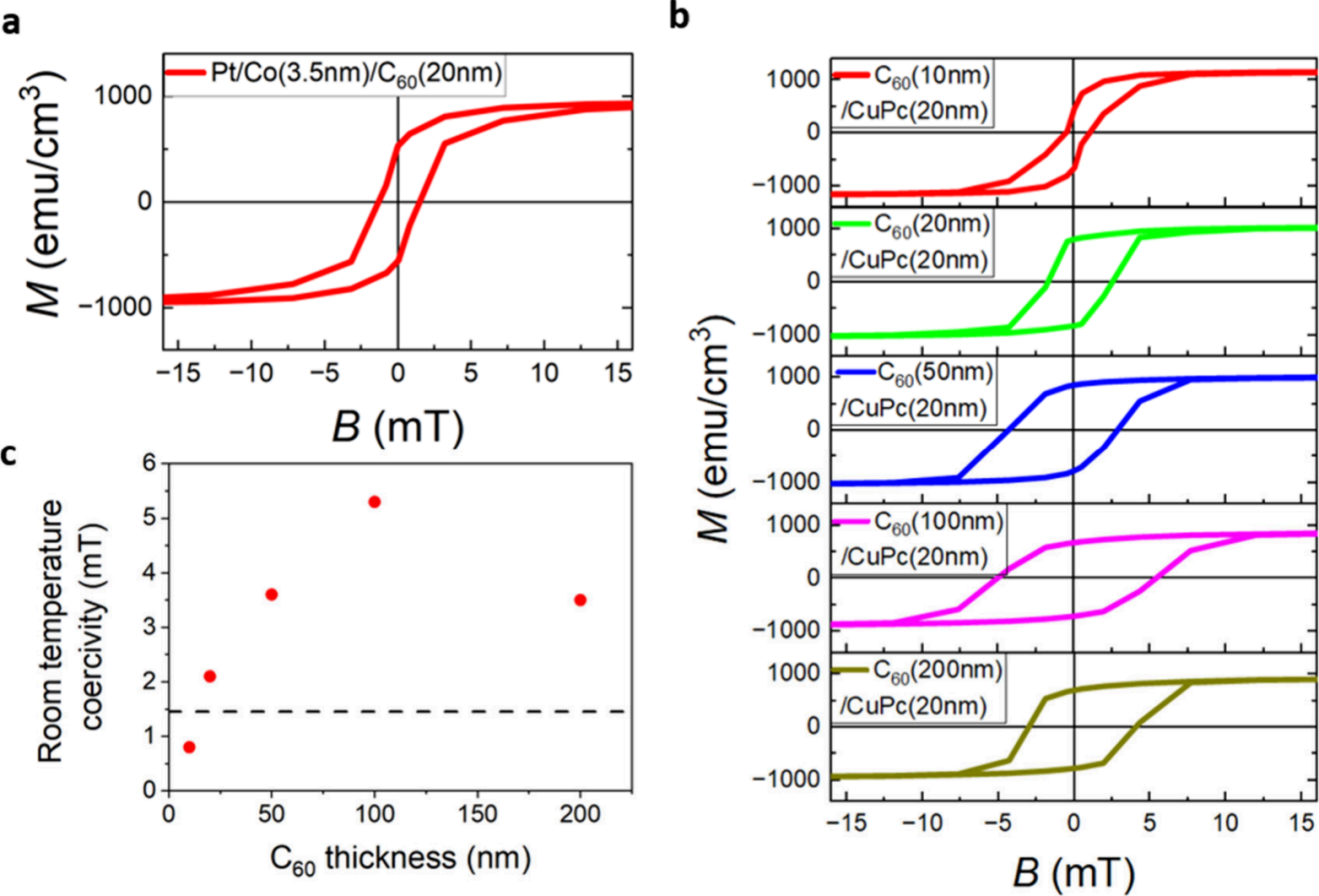
Room-temperature electric-field-induced coercivity tuning measured on a capped Pt/Co (3.5 nm)/C_60_ (*t* nm) interface utilizing a 20 nm CuPc layer. (a) Room-temperature magnetization curve measured on a Pt/Co (3.5 nm)/C_60_ (20 nm) interface. (b) Varying coercive fields of Co (3.5 nm) measured as its interface with varying C_60_ thicknesses in C_60_ (*t* nm)/CuPc (20 nm) acceptor–donor junctions, showing a room-temperature magnetic hardening effect, with coercivity maximized at a 100 nm thick C_60_ film (purple curve) and an exchange-bias-like shift measured for a 50 nm C_60_ thickness film. (c) Plot of the modified coercivity at room temperature at Pt/Co (3.5 nm)/C_60_ (*t* nm)/CuPc (20 nm) interfaces as a function of the C_60_ layer thickness, with the dashed line being coercivity for a reference Pt/Co (3.5 nm)/C_60_ (20 nm) film.

The presence of a built-in electric field at the molecular acceptor–donor heterojunction increases the coercivity compared to that of a reference Pt/Co (3.5 nm)/C_60_ (20 nm) sample (Figure 5c, dashed line). This coercivity enhancement is maximized for 100 nm separation between the molecular heterojunction and the metallo-molecular interface. We find that, in contrast to what is observed at low temperatures, the 200 nm C_60_ separation interface exhibits larger coercivity than a reference Pt/Co (3.5 nm)/C_60_ (20 nm) interface. We attribute this to the temperature-dependent nature of both the Debye screening and the intrinsic carrier density of C_60_ molecular layers. Data demonstrate up to around a factor of 3.5 enhancement of room-temperature coercivity compared to a reference Pt/Co (3.5 nm)/C_60_ (20 nm) interface by using the electric field intrinsically generated at C_60_ (*t* nm)/CuPc (20 nm) acceptor–donor junctions. Within the thickness-dependent series, a change in coercivity by a factor of up to 6 is measured as a function of the distance between the Co surface and the molecular diode interface (C_60_/CuPc), generating the built-in electric field. We find that this effect is not limited to Pt/Co (3.5 nm)/C_60_ (*t* nm)/CuPc (20 nm) interfaces, with similar tunability of room-temperature coercivity measured on Pt/Co (3.5 nm)/C_60_ (*t* nm)/MnPc (20 nm) and Pt/Co (3.5 nm)/C_60_ (*t* nm)/H_2_Pc (20 nm) structures; see Figures S10 and S11.

In conclusion, almost identical magnetic effects are observed in Co hybridized with H_2_Pc, C_60_, or CuPc molecules. Our results provide evidence that changes to the coercivity, remanence, magnetization, and loop asymmetry are due to a π-orbital hybridization-induced enhanced exchange interaction in Co and spin-polarized charge transfer at the interface. The results demonstrate that it is possible to modulate the magnetic properties of the “spinterface” through an internally generated or built-in electric field, utilizing C_60_/CuPc acceptor–donor (or CuPc/C_60_ donor–acceptor) heterojunctions. We have shown that, at low temperatures, the molecular magnetic hardening and pinning effects can be attenuated through this built-in electric field as well as changes to the saturation magnetization due to spin-polarized charge transfer. A finite low-temperature exchange bias emergence of up to 85 mT at 10 K has been measured on the Pt/Co (3.5 nm)/C_60_ (*t* nm)/CuPc (20 nm) interface, with *t* = 40, 50, or 100 nm depending on the sample batch, which we attribute to an enhanced Rashba coupling constant due to the electric field at the C_60_/CuPc interface. Utilizing the interface dipole of C_60_ (*t* nm)/CuPc (20 nm) structures, a built-in electric-field-induced manipulation of the room-temperature coercivity of the Co (3.5 nm)/C_60_ interface has also been demonstrated. The observed effects open new avenues of research for the design of metallo-molecular magnetic switches where the magnetization, coercivity, and remanence can be tuned via electric fields.

## Supplementary Material



## Notes

Additional details on experimental methods, STEM, X-ray reflectivity, and Raman spectroscopy characterization and further magnetometry data on Co interfaces of C_60_/MnPc, C_60_/H_2_Pc, as well as CuPc/C_60_ heterojunctions (PDF)
