# Supplementary material for: Acceptor–Donor Molecular Heterojunction Control of π‑Orbital-Induced Magnetic Properties of a 3d Ferromagnet
Source: Nano Lett. 2025 Aug 20;25(35):13394–400. doi: 10.1021/acs.nanolett.5c03762 (PMC12412173; doi:10.1021/acs.nanolett.5c03762)
Supplement: Supplementary file 1 [file nl5c03762_si_001.pdf]

Supporting Information

Acceptor-donor molecular heterojunction control of π-orbital induced magnetic properties of a 3d ferromagnet

Servet Ozdemir[†]*, Matthew Rogers[†], Zabeada Aslam[‡], Mannan Ali[†], Gilberto Teobaldi[ª], Timothy Moorsom[‡], B J. Hickey[†], Oscar Cespedes[†]*

[†] *School of Physics and Astronomy, University of Leeds, Leeds, UK*

[‡] *School of Chemical and Process Engineering, University of Leeds, UK*

[ª] *Scientific Computing Department, STFC, UKRI, Rutherford Appleton Laboratory, Didcot, UK*

*o.cespedes@leeds.ac.uk

*s.ozdemir@leeds.ac.uk

1. Experimental Methods

Thin film growth : Thin-film structures were grown on 0.65 mm thick c-plane sapphire films. The (111) textured Pt layers of ≈4 nm thickness were grown at 500 °C with e-beam evaporation at a growth rate of ≈0.1 Å/s. The substrate was than cooled down to room temperature and the 3.5 nm Co-layer was grown also with e-beam evaporation technique at a rate of ≈0.1 Å/s. Within the same chamber, organic molecule layers were sublimed onto the Co surface at a pressure of $\approx 5 \times 10^{-10}$ mbar with a rate of ≈0.3 Å/s and until the nominal thickness was measured on a quartz monitor. The cap layer was magnetron sputtered on top of the thin film structure, with the material used for capping being Nb or Cu with a thickness of $\approx$ 15 nm. Films were then structurally characterised using x-ray reflectivity and Raman spectroscopy as well as 4D-STEM.

Magnetometry: Magnetisation measurements were carried out using a SQUID-VSM magnetometer (MPMS3 from Quantum Design) which offers a resolution over 10^{-8} emu accompanied by temperature control with 50 mK stability.

4D-STEM/EDX: Electron transparent lamellae were prepared on a Ga ion Focussed Ion Beam (FIB). Cross sections were then measured in a Tescan Tensor Analytical Scanning Transmission Electron Microscope at 100 kV and a current of 250 pA. EDX spectra were recorded with a probe size of 0.4 nm. Cu signal was normalised to total x-ray intensity to correct for secondary excitation in holder. O K-emission is normalised to metal K-emission due to overlap at the edges. 4DSTEM was recorded with a 0.4 nm probe size and 6 mrad convergence. Maps were created by masking the diffraction patterns associated with the substrate and C_{60} lattice and rebuilding the image from the total integrated intensity.

2. X-ray reflectivity characterisation of the films

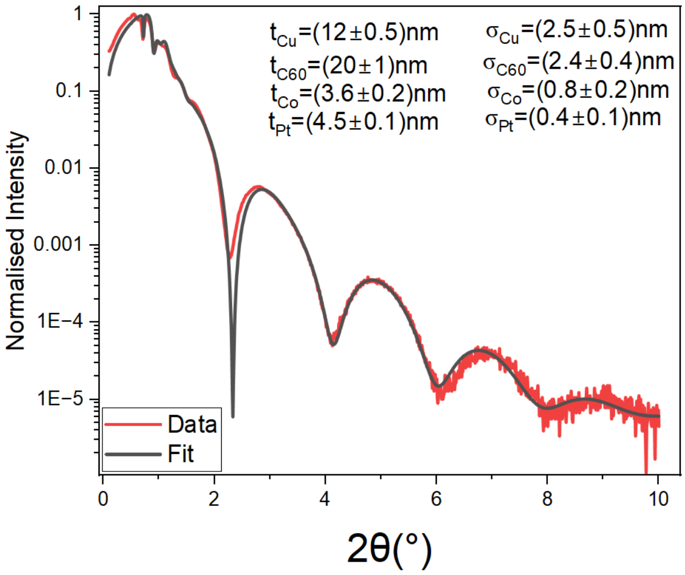

Figure S1. X-ray reflectivity and fitted model on a Pt/Co/C_{60}/Cu heterostructure, yielding a Co thickness of 3.6±0.2 nm.

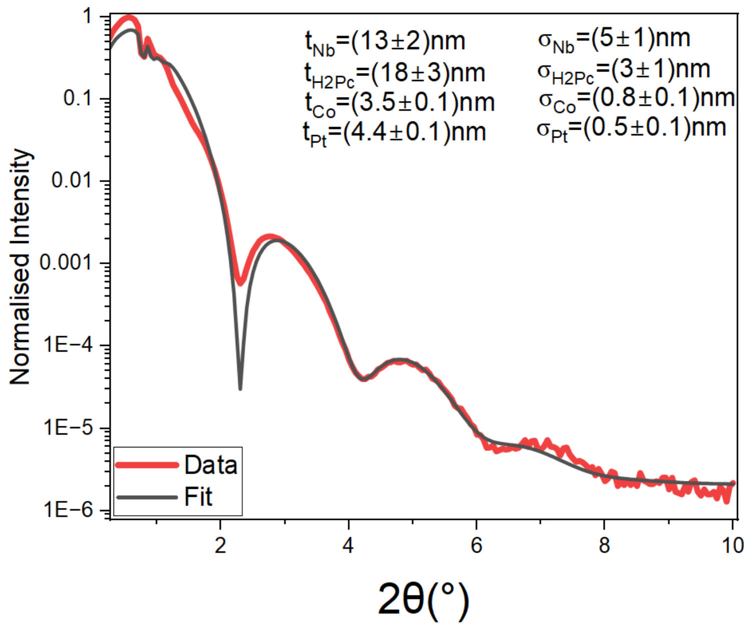

Figure S2. X ray reflectivity and fitted model on a Pt/Co/H_2Pc/Nb heterostructure, yielding Co thickness of 3.5±0.1 nm.

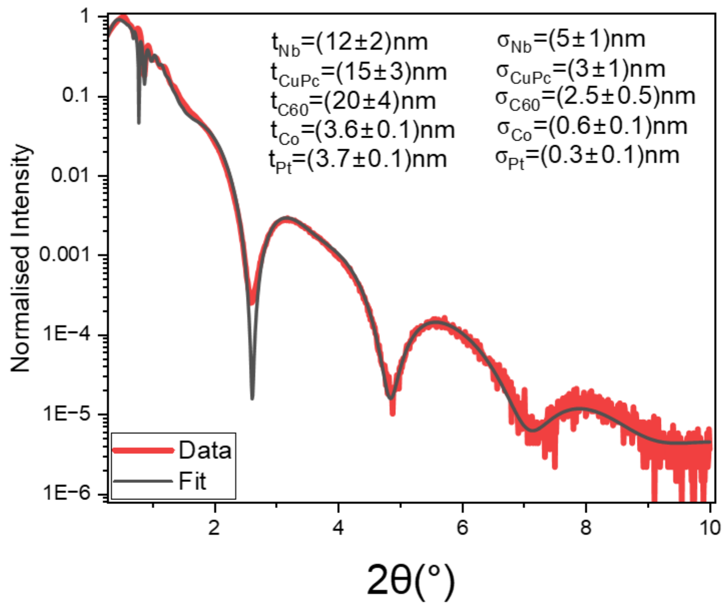

Figure S3. X-ray reflectivity and fitted model on a Pt/Co/C_{60}/CuPc/Nb heterostructure, yielding a Co thickness of 3.6±0.1 nm.

3. Raman Spectroscopy of a Pt/Co/C60/CuPc/Cap structure

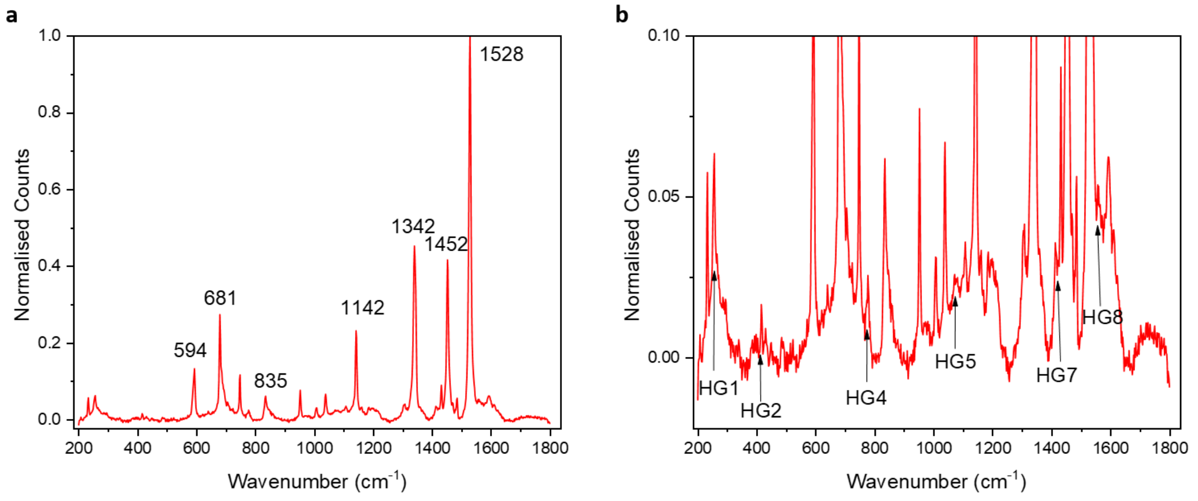

Figure S4. (a, b) Raman spectrum measured on a Pt/Co/C_{60}(20 nm)/CuPc(20 nm)/Nb structure with characteristic peaks corresponding to CuPc being more prominent as the top layer as well as lower intensity peaks (b) corresponding to C_{60}. A 532 nm laser was used with a grating of 600 lines/mm.

5. Magnetometry curve on Pt/Co/C60(200nm)/CuPc/Cap structure

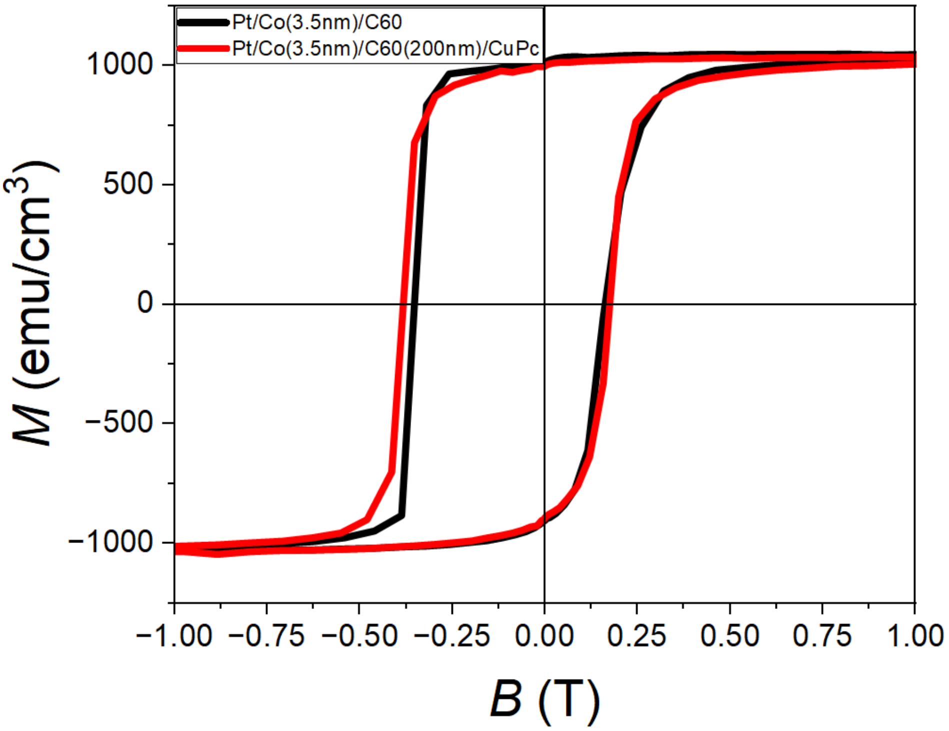

Figure S5. Magnetometry curves for a reference Cu-capped Pt/Co(3.5 nm)/C_{60}(20 nm) heterostructure and a Nb-capped Pt/Co(3.5 nm)/C_{60}(200 nm)/CuPc(20 nm) heterostructure show the same saturation magnetisation.

6. Magnetometry on Pt/Co/C60/MnPc/Cap structures –

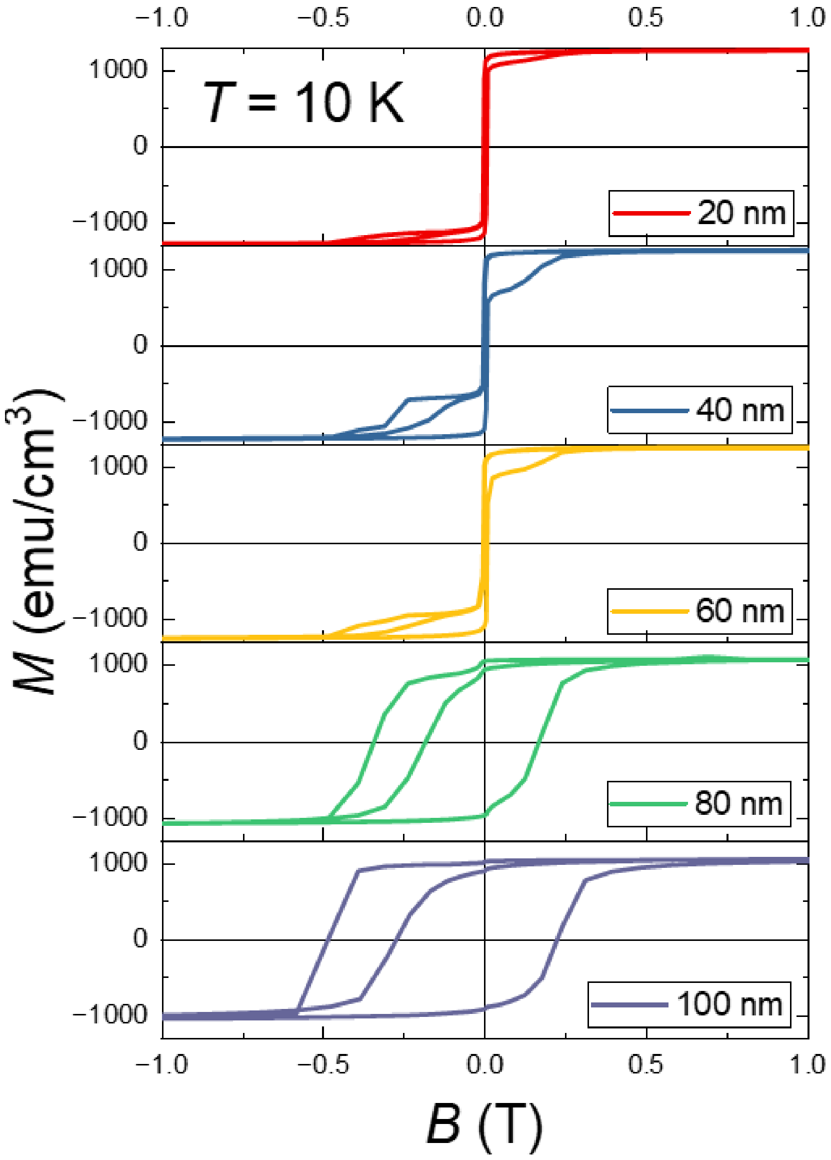

Figure S6. Magnetometry data for Nb-capped Pt/Co(3.5 nm)/C_{60}(t (nm))/MnPc(20 nm) structures, showing attenuation of hybridisation effects at Co(3.5 nm)/C_{60}(t (nm)) interface as a function of the C_{60} layer thickness, t. Magnetic hardening and pinning is restored for Pt/Co(3.5 nm)/C_{60}(80nm)/MnPc(20 nm) and Pt/Co(3.5 nm)/C_{60}(100 nm)/MnPc(20 nm) samples. There is a small exchange bias onset on Pt/Co(3.5 nm)/C_{60}(100 nm)/MnPc(20 nm), with an offset field of $\approx$26 mT.

7. Magnetometry on Pt/Co/C60/H_2Pc/Cap structures –

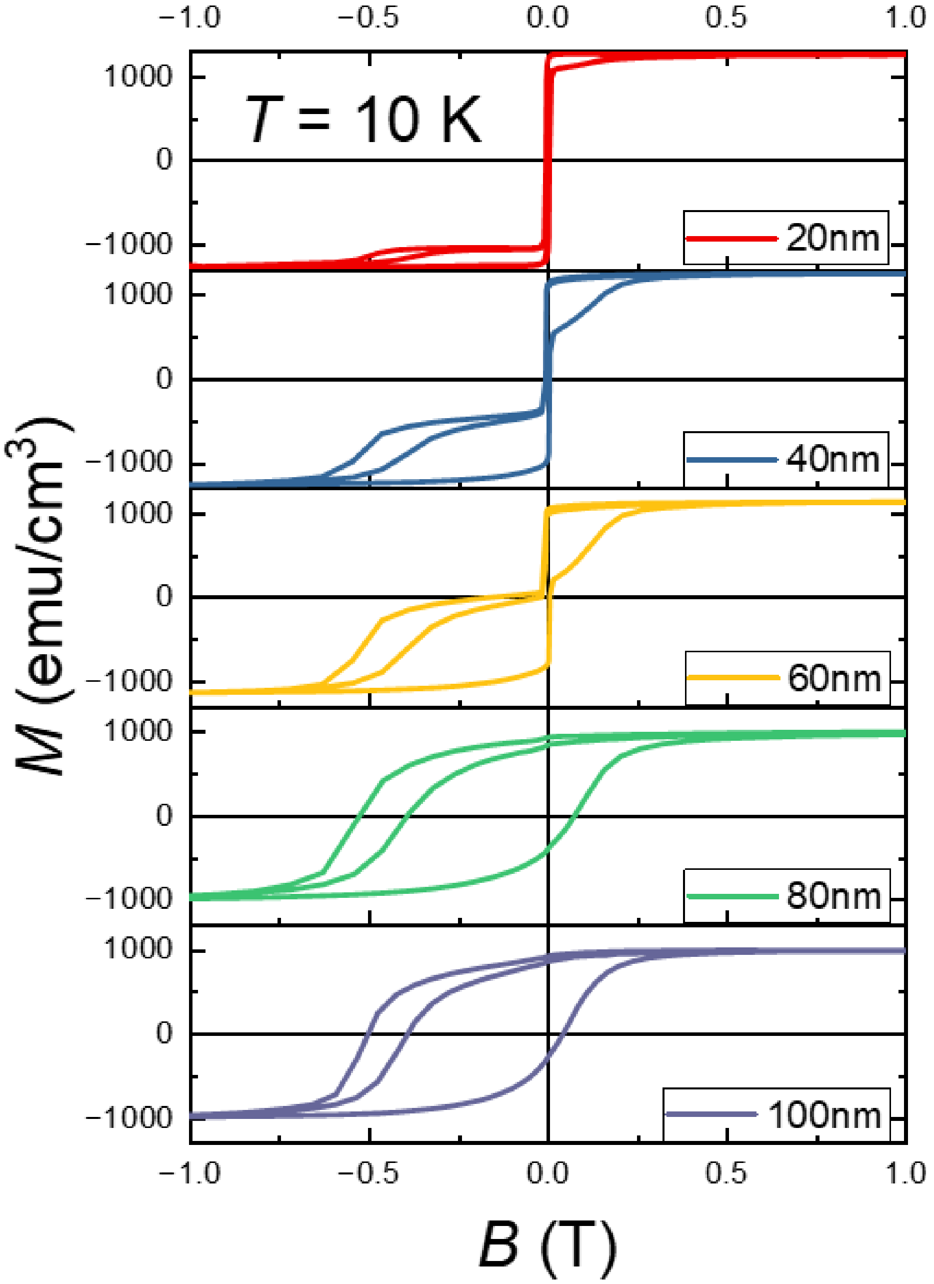

Figure S7. Magnetometry data for Nb-capped Pt/Co(3.5 nm)/C_{60}(t (nm))/H_2Pc(20 nm) structures, showing attenuation of hybridisation effects at Co(3.5 nm)/C_{60}(t (nm)) interface as a function of C_{60} layer thickness, t. As it was the case with MnPc and CuPc, magnetic hardening and pinning is restored for $t \gtrsim 80\,nm$, in Pt/Co(3.5 nm)/C_{60}(80 nm)/H_2Pc(20 nm) and Pt/Co(3.5 nm)/C_{60}(100 nm)/H_2Pc(20 nm) samples, where there is also an exchange bias. The offset bias has values of ≈160 mT and ≈176 mT, respectively, for the two structures.

8. Magnetometry on Pt/Co/CuPc/C60/Cap structures

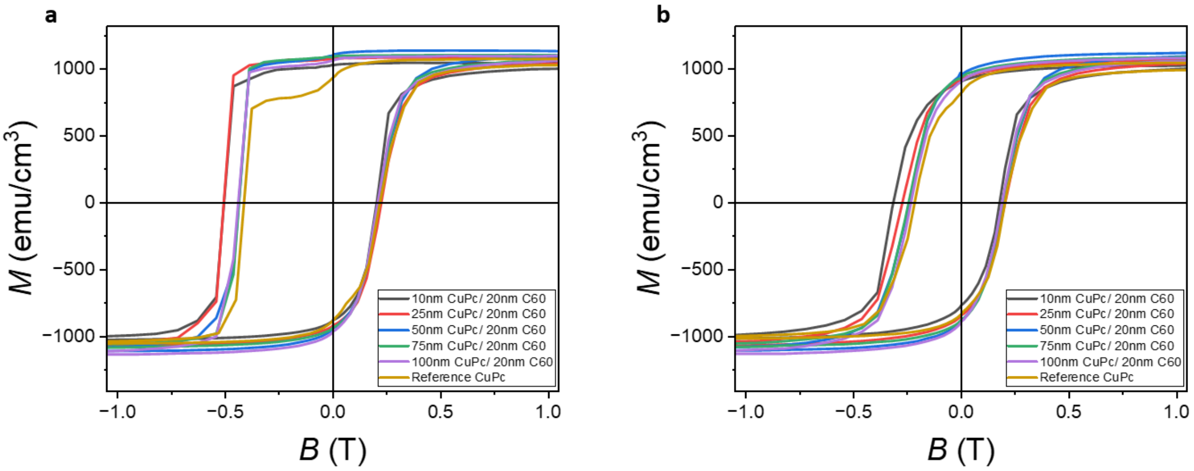

Figure S8. Magnetic hysteresis curves measured on Cu-capped Pt/Co(3.5nm)/CuPc(*t*)/C_{60}(20 nm) interfaces a) measured post field cooling at 2 T suggesting an enhancement of the coercivity measured at first reversal point, and b) training curves showing the reduced bias.

9. Aging effects for Cu and Nb capped samples

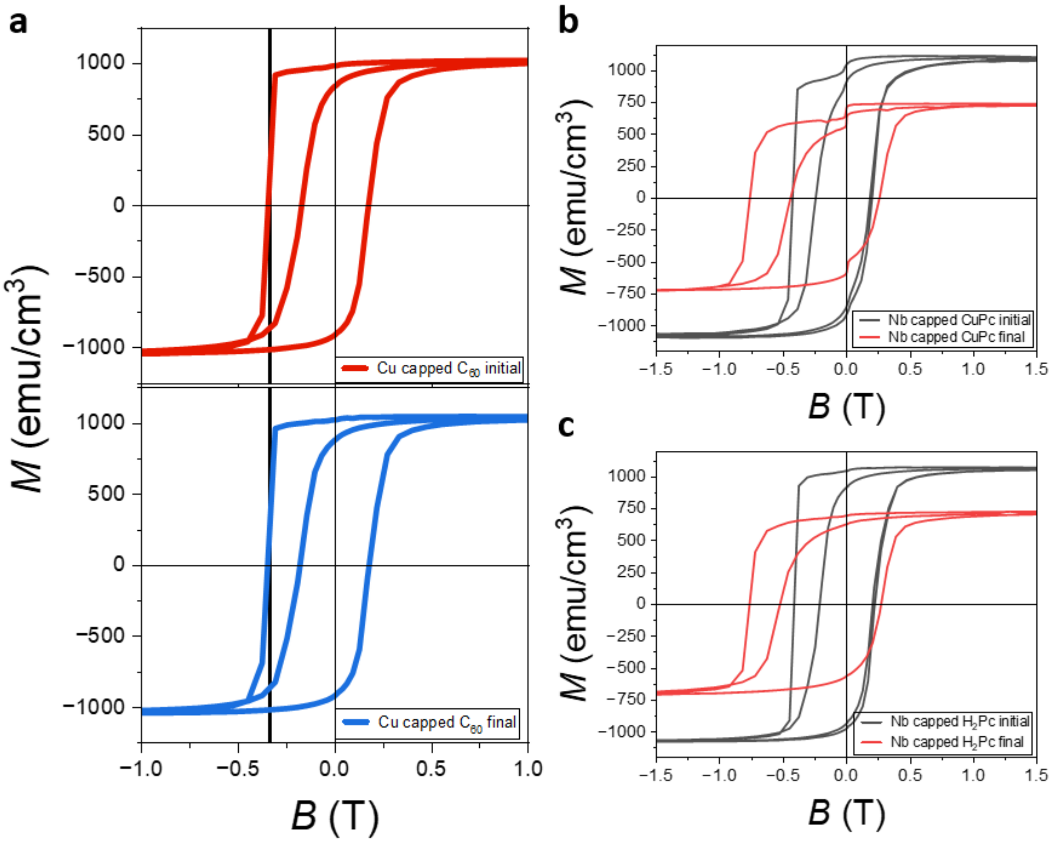

Figure S9. Aging effects measured on differently capped metallo-molecular structures at T = 10 K. a) Magnetic hysteresis measurements carried on Pt/Co(3.5 nm)/C_{60}(20 nm)/Cu(15 nm) heterostructure. The time gap between initial (upper panel) and final (lower panel) was 6 months, suggesting no degradation effects on Cu capped samples. b) Magnetic hysteresis curves measured on Nb-capped Pt/Co(3.5 nm)/CuPc(20 nm)/Nb(15 nm), with the initial measurement post sample growth being the black curve, and the final measurement six months later in red. The result shows a reduced magnetisation and an enhanced magnetic hardening that we attribute to oxidation. c) Magnetic hysteresis curves measured on Nb capped Pt/Co(3.5 nm)/H_2Pc(20 nm)/Nb(15 nm) structure with the initial measurement post sample growth again the black curve, and the final measurement six months later in red. The measurement shows once more a reduced magnetisation and an enhanced magnetic hardening that we attribute to oxidisation.

10. Debye screened electric field

Debye screened electric field across a slab of C_{60} film can be modelled as

$$E(z) = \frac{\sigma}{2\varepsilon_0\varepsilon_r} \exp\left(\frac{-z}{\lambda_D}\right)$$

where σ is the sheet carrier density at the C_{60}/CuPc interface, which has been calculated to be[1] on the order of 10^{12} cm^{-2}, ε_0 is the permitivity of free space, ε_r is the relative permitivity of C_{60} film (taken to be 4), z is the thickness of the C_{60} film, and λ_D is the Debye screening length. Debye screening length λ_D is estimated to be 38 nm with Debye-Huckel length equation using the intrinsic carrier densities determined in C_{60} films[2,3] on the order of 10^{15} cm^{-3}.

11. Room temperature magnetometry on Pt/Co/C60/MnPc/Cap structures

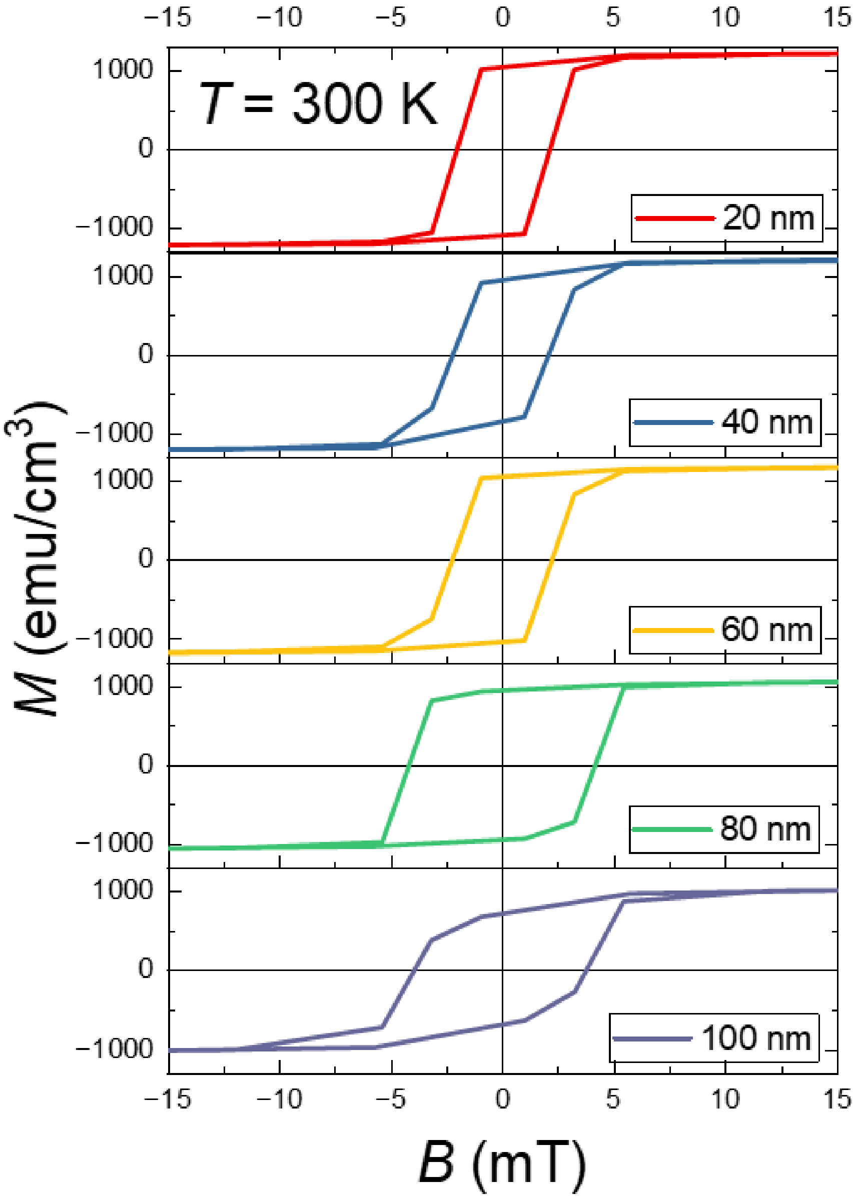

Figure S10. Room temperature electric field induced coercivity tuning measured on Nb-capped Pt/Co(3.5 nm)/C_{60}(t nm)/MnPc(20 nm) structures.

12. Room temperature magnetometry on Pt/Co/C60/H2Pc/Cap structures

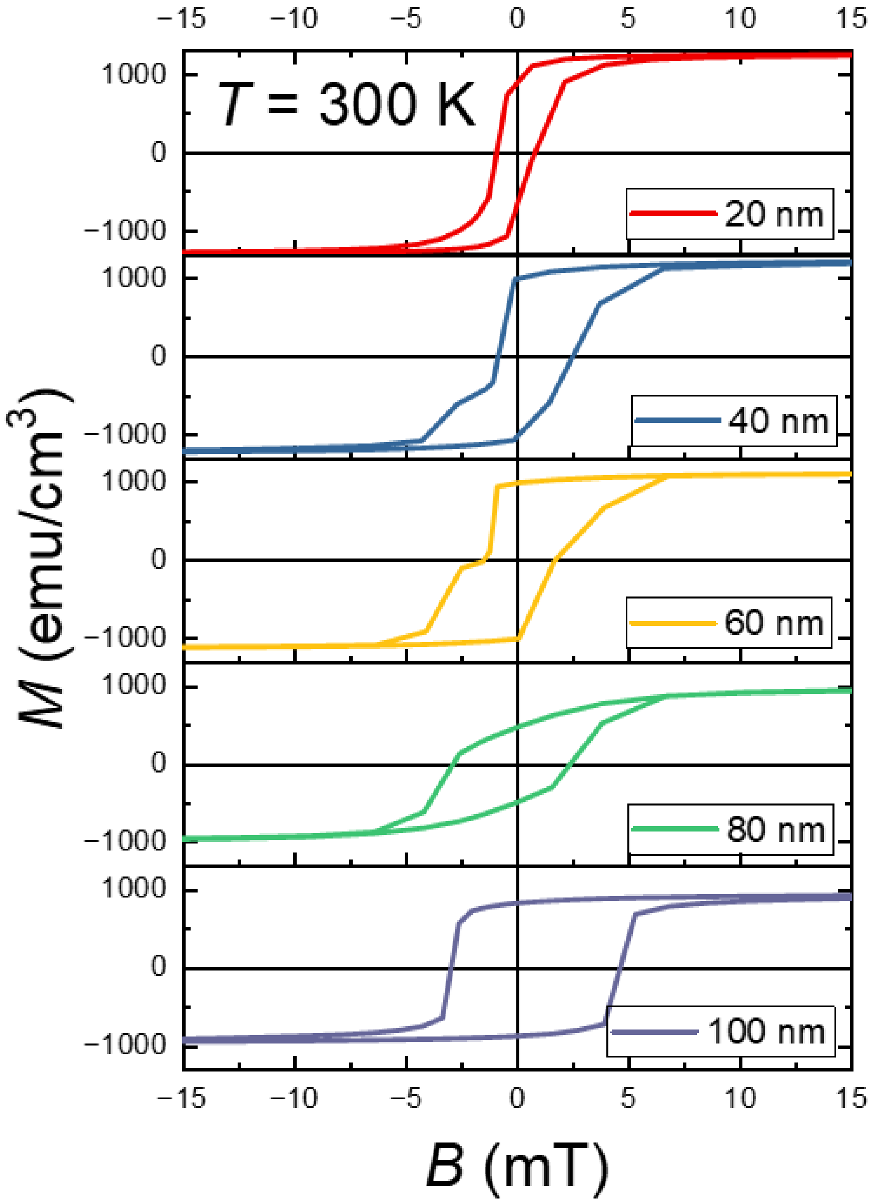

Figure S11. Room temperature electric field induced coercivity tuning measured on Nb-capped Pt/Co(3.5 nm)/C_{60}(t nm)/H_2Pc (20 nm) structures.

10. 4D-STEM of Pt/Co/C60/CuPc/Nb molecular diode structure

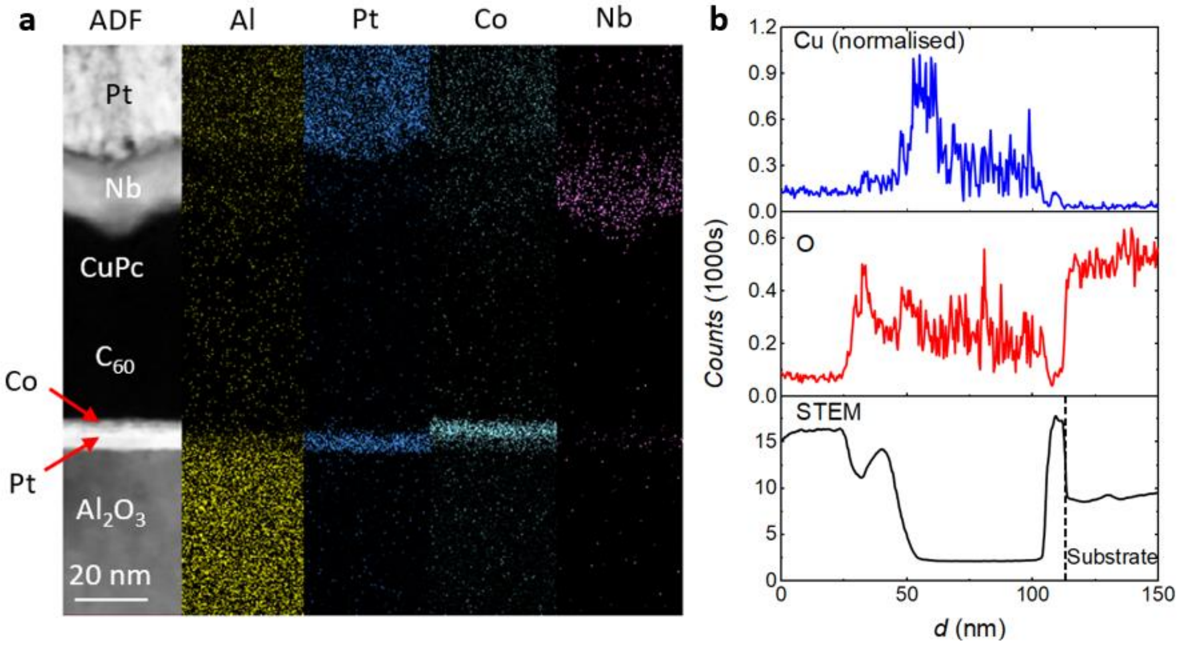

Figure S12. Scanning transmission electron microscopy (STEM) characterisation of a Pt/Co/C_{60}/CuPc/Nb molecular acceptor-donor heterointerface structure. (a) Annular dark field (left, labelled ADF) and energy dispersive x-ray spectroscopy (EDX) images of the samples for depicted elements (Al - green, Pt - blue, Co-turquoise and Nb -purple). (b) Profiles across EDX images for Cu (blue) and O (red) elements as well as the STEM signal (black) depicting the substrate boundary with a dashed line.

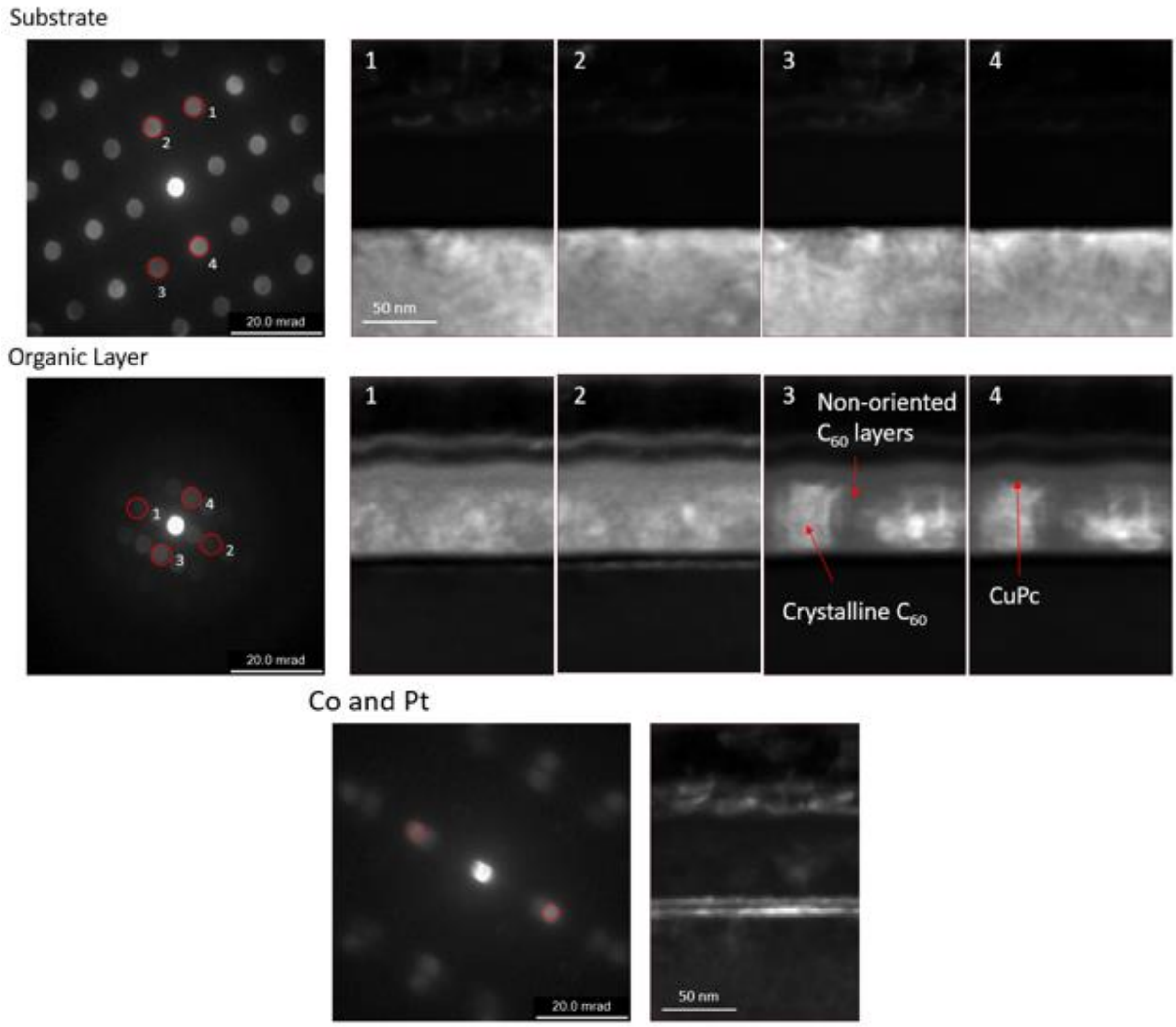

Figure S13. 4D-STEM images of a Pt/Co/C_{60}/CuPc/Nb molecular acceptor-donor heterointerface structure, with corresponding images obtained for diffraction spots on the substrate, organic layer, and Co/Pt metallic layer as per the figure labels.

11. Computational Methods

Collinear van der Waals (vdW) corrected DFT simulations were carried out in the Projected Augmented Wave (PAW) formalism as implemented in the VASP program.[4] We used the PBE approximation[5] to the exchange-correlation (XC) functional, a 400 eV plane-wave energy cut-off, (0.2 eV, 1st order) Methfessel-Paxton electronic smearing,[6] and a Γ-centered k-point grid of 5 for hexagonal C_{60}/Co(0001)-4x4 slab. For C_{60}/Co(0001)-4x4, we used the minimum energy geometry in ref 7. vdW corrections were applied following Grimme's parameterization.[8] Bader charge analysis[9] was carried out based on the total charge density, accounting for both the electronic and ionic core charges. All the simulations were carried out including dipole corrections as available in VASP, direct calculation of dipole densities (μ_σ). The electrostatic potential step (vacuum-level shift), Δ, associated with a given dipole density can be calculated in a planar dipole layer approximation[10] as (atomic unit): $\Delta=4\pi\mu_\sigma$.

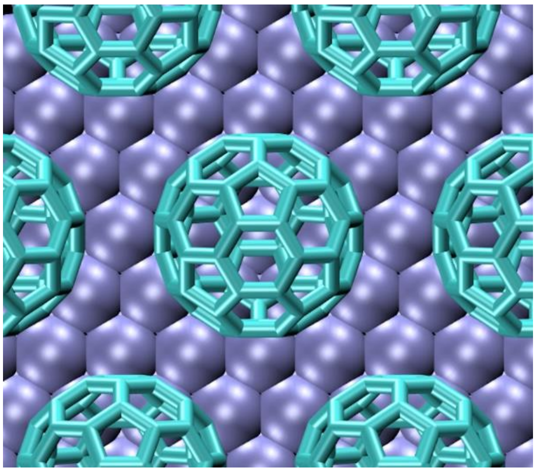

Figure S14. Optimised model for the hexagonal C_{60}/Co(0001)-4x4 slab used to compute interface dipole value reported in the main manuscript. Co: violet, C: cyan.

References

1. Sai, N. *et al.* Understanding the interface dipole of copper phthalocyanine (CuPc)/C 60: Theory and experiment. *Journal of Physical Chemistry Letters* **3**, 2173–2177 (2012).

2. Hoshimono, K., Fujimori, S., Fujita, S. & Fujita, S. Semiconductor-Like Carrier Conduction and Its Field-Effect Mobility in Metal-Doped C60 Thin Films. *Jpn J Appl Phys* **32**, 1070–1073 (1993).

3. Li, F. *et al.* Acridine orange base as a dopant for n doping of C60 thin films. *J Appl Phys* **100**, (2006).

4. Kresse G. and Furthmüller J., Efficient iterative schemes for ab initio total-energy calculations using a plane-wave basis set. *Physical Review B* **54**, 11169, (1996).

5. Perdew J. P., Burke K., and Ernzerhof M., Generalized gradient approximation made simple. *Physical Review Letters*, **77**, 3865, (1996).

6. Methfessel M. and Paxton A.T, High-precision sampling for Brillouin-zone integration in metals. *Physical Review B*, **40**, 3616, (1989).

7. Moorsom T. et al., Spin-polarized electron transfer in ferromagnet/*C*60 interfaces. *Physical Review B* , **90**, 125311, (2014).

8. Grimme S., Semiempirical GGA-type density functional constructed with a long-range dispersion correction. *Journal of Computational Chemistry*, **27**, 1787, (2006).

9. Henkelman G., Arnaldsson A., Jonsson H., A fast and robust algorithm for Bader decomposition of charge density. *Computational Materials Science*, **36**, 354 (2006**).**

10. J. Junquera, M. H Cohen, K. M. Rabe, *J. Phys. Condens. Matt.* **19**, 213203 (2007).
